# Gaze communicates both cue direction and agent mental states

**DOI:** 10.3389/fpsyg.2024.1472538

**Published:** 2024-09-30

**Authors:** Florence Mayrand, Francesca Capozzi, Jelena Ristic

**Affiliations:** ^1^Department of Psychology, McGill University, Montreal, QC, Canada; ^2^Department of Psychology, Université du Québec à Montréal, Montreal, QC, Canada

**Keywords:** gaze direction, perspective taking, cue direction, mental content, theory of mind

## Abstract

**Introduction:**

Although it is well established that humans spontaneously attend to where others are looking, it remains debated whether this gaze following behavior occurs because gaze communicates directional information (i.e., *where* an agent is looking) or because gaze communicates an agent’s inferred mental content (i.e., *what* an agent perceives), both of which rely on the processes involved in the general Theory of Mind ability.

**Methods:**

To address this question, in two Experiments we used a novel task to measure how spatially dissociated and spatially combined effects of an agent’s gaze direction and perceived mental content influence target performance. We also contrasted performance for social directional cues and nonsocial arrows.

**Results:**

Our data revealed that performance was compromised when cue direction and mental content dissociated relative to when they combined. Performance for dissociated components was especially prominent when a social avatar served as a cue relative to a comparison arrow.

**Discussion:**

Together, these data show that a typical gaze signal communicates information about both *where* an agent is attending and *what* they are attending to.

## 1 Introduction

Visual information conveyed by gaze (eyes, head, or body deviation) enables quick communication of social messages (Capozzi and Ristic, 2018). As such, the ability to follow gaze has been implicated in both basic social functions like gaze following and joint attention (Frischen et al., 2007) as well as more complex social behaviors such as attitude formation (Toscano et al., 2018) and social status inference (Capozzi et al., 2019). Strikingly however, the large body of research on gaze following remains agnostic on the nature of messages conveyed by gaze (Capozzi and Ristic, 2020; Frischen et al., 2007). Specifically, there remains a key outstanding question of whether humans follow gaze because it conveys directional information about *where* the items of a gazer’s interest are located or because gaze also coveys mentalistic information about *what* the gazer perceives (Capozzi and Ristic, 2020). Using a novel behavioral test procedure, here we dissociate the contributions of directional and mentalistic components of the gaze signal and in doing so, show that decoupling of this information leads to the largest detriments in performance. This suggests that ordinarily gaze communicates *both* directional information about where the gazer is looking and mentalistic information about *what* the gazer is perceiving.

The nature of signals communicated by gaze has been the subject of a longstanding debate (e.g., Capozzi and Ristic, 2020). The proponents of the directionality account argue that spontaneous following of gaze, often experimentally demonstrated by the gaze cuing procedures (Friesen and Kingstone, 1998) or dot perspective task (Samson et al., 2010), reflects the influence of the cue’s direction indicating where in space the gazer’s attention is directed (Cole and Millett, 2019; Santiesteban et al., 2017). In contrast, the proponents of the mentalizing account maintain that this behavior is instead driven by the spontaneous adoption of the gazer’s visual perspective, which aids with observers inferring and sharing the representation of the gazed-at object with the gazer (Apperly and Butterfill, 2009). Supporting the directionality account, gaze following has been found to occur similarly regardless of whether it is elicited by the direction of social gaze or a nonsocial cue (e.g., an arrow; Kingstone et al., 2019). Supporting the mentalizing account, gaze following magnitude is reduced when participants believe gaze cues are delivered by a randomized computer sequence (Wiese et al., 2012) or a person whose line of sight is obstructed (Baker et al., 2016).

Detecting and understanding gaze signals is often understood as a key precursor and a component of Theory of Mind, which generally denotes one’s ability to take the mental perspective of others (Premack and Woodruff, 1978; Baron-Cohen, 1991). Within this context, directional and mentalistic aspects of gaze track with two proposed levels of mental perspective taking complexity, thus offering insights into how each of the gaze processing aspects may inform the Theory of Mind (ToM). Specifically, the directional gaze account appears to align with the processes associated with Level-1 visual perspective taking. This aspect of gaze communication requires the observer to understand the physical orientation of the gaze in others, which is foundational in both social cognition and ToM (Kessler and Rutherford, 2010). Crucially, Level-1 visual perspective taking involves the ability to comprehend whether an object is visible within the line of sight of another person, regardless of whether it is visible from one’s own line of sight (Flavell et al., 1981). Comparatively, the mentalizing gaze account appears to align with the processes associated with Level-2 visual perspective taking, in which one understands the visual aspects of a scene relative to an imagined viewpoint of a gazer, such that one is able to understand that even if an object is visible to the self and another person, this does not mean they share the same mental representation (Flavell et al., 1981). This requires representation of the gazer’s mental states thereby invoking a deeper level of mental state attribution (Todd et al., 2017). To maintain connection with the existing research and the ongoing dialogue between directional and mentalistic accounts (e.g., Capozzi and Ristic, 2020), here we refer to the two processes implicated in gaze signals as the directional component of gaze and the mentalistic component of gaze, whereby the directional component is conceptualized as reflecting the influence of gaze cue direction on behavior (i.e., Level-1 perspective taking) while the mentalizing component is conceptualized as reflecting the influence of the understanding of the gazer’s mental perspective on behavior (i.e., Level-2 perspective taking).

**Figure 1:**
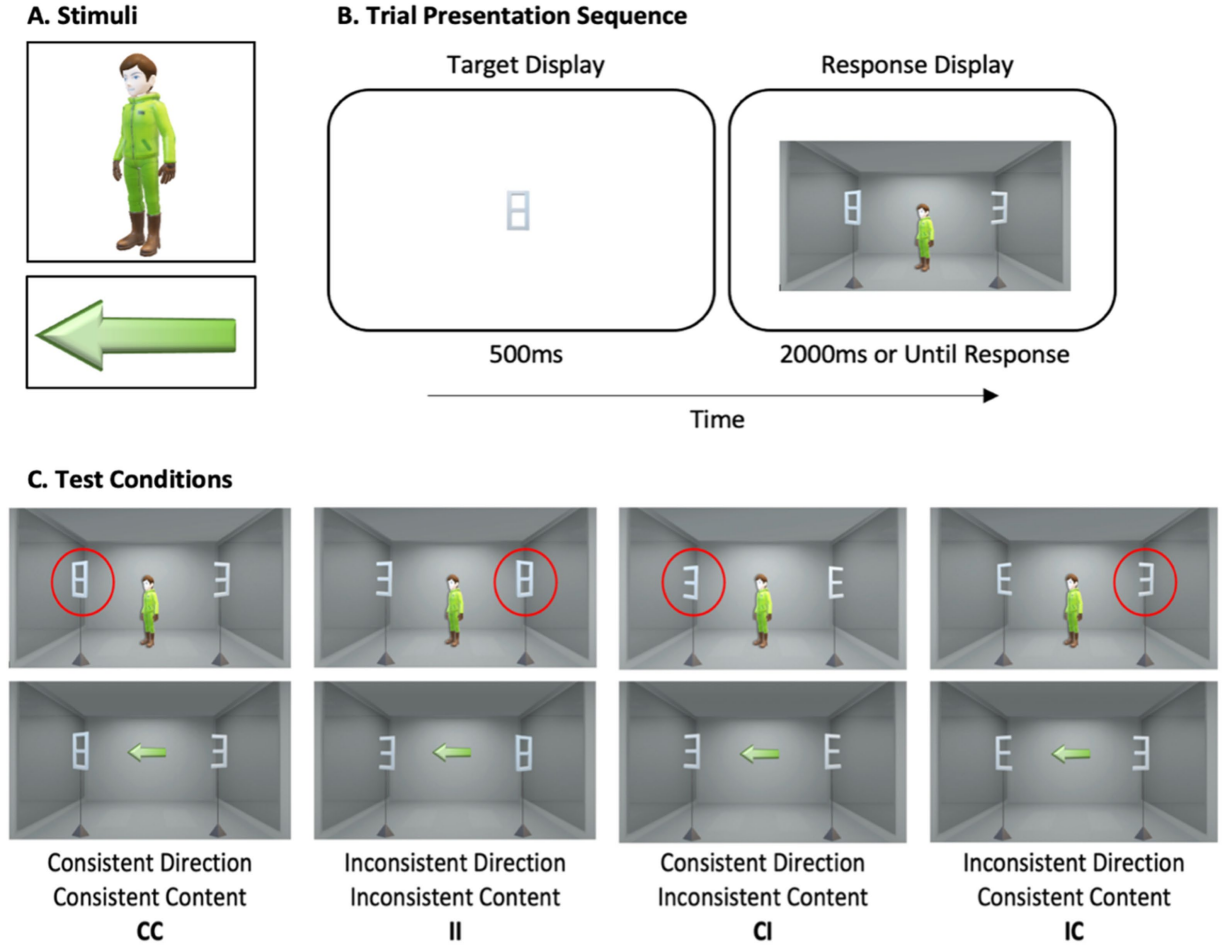
Stimuli, example stimulus presentation sequence, and test conditions. **(A)** Illustration of cue and target stimuli. **(B)** Example trial sequence. Participants were first presented with an image of the trial response target for 500 ms. The response display was then presented for 2000 ms or until participants responded. **(C)** Illustration of the four test conditions for each cue type. Target is highlighted for the reader with a red circle. Stimuli are not drawn to scale.

To investigate the contribution of each of these components in gaze following, we designed a novel behavioral task in which we measured the combined and dissociated effects of these two processes on target performance. We ran two experiments in which participants were presented with an image of a central cue (a human avatar or an arrow) and were asked to localize a peripheral target that was flanked by a non-target distractor (Figure 1). Providing directional information to the observer, the cue indicated a left or right spatial location, that is, it either pointed toward the response target or toward a non-target distractor equally often. Providing mentalistic information, the avatar also “perceived” either same target as the observer or the non-target distractor due to our manipulation of targets which afforded multiple representations depending on the point of view. Critically, in the combined conditions, the directional and mentalistic processes were congruent, as they would be in a typical gaze signal, such that the cue both indicated the target directionally the observer gazed to and mentally perceived its content from its perspective. Critically, in the dissociated condition, they could directionally look at the same target identity as the observer, but they may nevertheless perceive a different object. Similarly, the cue may also look at a different target identity as an observer.

Two Experiments were run. They were identical except that in Experiment 1 social gaze and nonsocial avatar were manipulated between subjects while in Experiment 2 the two cues were manipulated within participants. Experiment 2 thus also provided a direct replication of the initial proof of concept obtained in Experiment 1.

If gaze typically communicates information about both cue direction and mental content of the gazer, target performance should suffer the most when these two components dissociate relative to when they combine. That is, a condition in which the avatar is looking at a peripheral location containing the observer’s target but perceives a distractor from its own perspective should result in slower responses relative to a condition in which the avatar is looking at a peripheral location containing the observer’s target and perceives the same target from its own perspective. This is because in the former case, the directional and mentalistic information communicated by the avatar are spatially dissociated while in the latter case the two pieces of information are spatially combined as they would be in a typical gaze signal. Hence, when directional and mentalistic processes occur together in a congruent manner (i.e., the avatar is both looking at and perceiving the same target as the observer from its visual perspective) performance is facilitated. To understand whether any these effects may be unique to directional cues delivered by social agents, we also subjected a nonsocial central arrow cues to the same test.

## 2 Experiment 1

While gaze is typically understood to reflect human social communication, directional cues such as arrows have often been used as a comparison (i.e., Santiesteban et al., 2014; Kingstone et al., 2019; Kuhn and Kingstone, 2009), due to their similar directional representation but lesser social value. In Experiment 1, we examined combined and dissociated contributions of the mentalistic and directional components of gaze elicited by a social avatar and nonsocial arrow cues. If gaze typically invokes both directional and mentalistic representations in observers, target-related response detriments for dissociated representations should be more pronounced when a social avatar serves as a cue relative to when a nonsocial arrow serves as cue.

### 2.1 Methods

#### 2.1.1 Participants

An *a priori* power analysis using an estimated moderate size estimate of *r* = 0.25 for the variability in the magnitude of the gaze cuing with mental state attribution (approximated from Moriguchi et al., 2006; Sulpizio et al., 2015; Tomei et al., 2017) indicated that data from about 95 participants would yield power of 0.8 and data from 130 participants would yield power of 0.9 (Alpha = 0.05). Data from 220 participants were included in the analysis^1^, with 111 participants randomly assigned to view the avatar cue (97 women,14 men, Mean age = 20.53 years, *SD* = 2.34) and 109 participants (92 women, 15 men, 2 other, Mean age = 20.67 years, *SD* = 2.82) randomly assigned to view the arrow cue. All procedures were approved by the University’s Research Ethics Board. Informed consent was obtained from all participants. Participants were recruited from the McGill University participant pool and compensated with course credit^2^. All participants reported native English fluency, no history of psychiatric or neurological conditions, and normal or corrected-to-normal vision. Deidentified data are available at osf.io/3xcqk.

#### 2.1.2 Apparatus and stimuli

Figure 1 illustrates the stimuli (A), sample trial sequence (B), and the four test conditions for each avatar and arrow cue (C). Cues were images of a human avatar and an arrow, which were equated for length (Figure 1A). The cues were positioned at fixation. Target and distractor stimuli, which were shown on the left and right of fixation respectively, were a letter E, a number 3, and a number 8 (Figure 1C). They were equated for size. Each response target, either 3, E, or 8, was always flanked by a unique distractor, creating three unique target-distractor combination (3 / E; 3 / 8; E / 8).

The study was administered online via Testable (https://www.testable.org/). The experiment was launched on participants’ personal computers. Because participants were alone in their own environments to complete the study, they were instructed to minimize distractions in their surroundings for the duration of the experiment. The entire stimulus response display image including all stimuli in proportions was scaled to approximately 50% of individual participants’ screens. The display target images were rendered in grayscale; the central cues were rendered in green.

#### 2.1.3 Design

The experiment was a mixed design, with *Cue Type* (Avatar; Arrow) included as a between subjects variable, and *Cue Direction Consistency* (Consistent; Inconsistent) and *Target Content Consistency* (Consistent; Inconsistent) included as within subjects variables.

*Cue Type* manipulated the type of the central cue and varied between an avatar and an arrow. Half of the participants were presented with an avatar cue and the other half was presented with an arrow cue. The cue assignment was randomized across participants.

*Cue Direction Consistency* manipulated whether the response target appeared at the spatial location directionally indicated by the cue (i.e., Consistent; Figure 1C) or at the opposite location (i.e., Inconsistent). This variable is a composite of cue direction and target location factors, each of which varied independently and equiprobably between left and right spatial positions.

*Target Content Consistency* manipulated the observed and inferred avatar’s mental content. The avatar’s mental content (i.e., what they would perceive from their viewpoint) was either consistent or inconsistent with the participant’s perception of target identity. Consistent target content between the observer and the avatar was induced using target stimuli that invoke the same percept from the observer’s and the avatar’s perspectives (i.e., number 8; Figure 1C). That is, the response target 8 would have the same percept from both the observer’s and the avatar’s perspective. Inconsistent target content between the observer and the avatar was induced using target stimuli that are perceived as a target from the observer’s perspective but a distractor from avatar’s perspective and vice versa. That is, for the avatar, the observer’s response target 3 would appear as a distractor letter E while the response target E would appear as a distractor number 3. Target identity and target location were intermixed, varied independently and equiprobably with cue direction. Each target-distractor combination appeared equally often.

The combination of these variables created four key conditions of interest. In the *Consistent direction/Consistent content (CC)* and the *Inconsistent direction/Inconsistent content (II)* conditions, cue direction and target mental content combined, such that both were either consistent (CC) or inconsistent (II). That is, the cue either pointed at or away from the target, which matched the observer’s and the avatar’s perspective. In contrast, in the *Consistent direction/Inconsistent content (CI)* and the *Inconsistent direction/Consistent content (IC)* conditions, cue direction and target mental content dissociated. In the CI condition, the avatar looked the response target but perceived a distractor (i.e., the observer’s response target 3 appears as a distractor E). In contrast, in the IC condition, the avatar looked at a distractor but perceived the target at that location, since the non-target distractor may be perceived as the target from the avatar’s perspective (i.e., distractor 3 appears as the response target E).

#### 2.1.4 Procedure

Example trial sequence is shown in Figure 1B. Trials began with an image of a response target (3, E, 8) for 500 ms to inform participants about the target they need to respond to for that trial. Then, the display showing the central cue and the target/distractor combination was presented. The display remained visible for 2000 ms or until participants responded. Participants were informed that the cue direction was not predictive of target location or target identity. They were asked to localize the target quickly and accurately by pressing the ‘b’ and ‘h’ keys on the keyboard. Target location (left, right) and key response (‘b’ or ‘h’) pairing was counterbalanced between participants.

The experiment consisted of four blocks of 80 trials, for a total of 320 trials. Each block contained 20 trials for each of the 4 test conditions (CC, II, CI, IC). All conditions were intermixed and presented equally often using a random sequence. Eight practice trials with performance feedback were run first. The study was completed in 38.85 min on average (SD = 9.13 min).

### 2.2 Results

Response anticipations (RT < 200 ms) and timeouts (RT > 1800s) accounted for 1.49% of all trials and were removed from analyses. Performance was high overall at 92.22%. Accuracy was examined across *Cue Type* (Avatar, Arrow; between-subjects) and the four test *Conditions* (CC, II, CI, IC; within-subjects) using a mixed-effects ANOVA. No speed-accuracy tradeoff was evident in the data, with CC (*M* = 0.96) and II (*M* = 0.96) conditions returning higher overall accuracy then CI (*M* = 0.89) and IC (*M* = 0.88) conditions, [*F* (2.30, 502.11) = 303.67, *p* < 0.001, MSE = 0.002, *ηp^2^ = 0*.58]. *Cue Type x Condition* interaction was not reliable, [*F*(3, 654) = 0.19, *p* = 0.90, *MSE* = 0.002, *ηp2* = 0.001]. When Mauchly’s test was significant, the Greenhouse–Geisser degrees of freedom are reported. All follow-up, *t*-tests two-tailed and Bonferroni corrected. Data were analyzed using SPSS 27.

**Figure 2:**
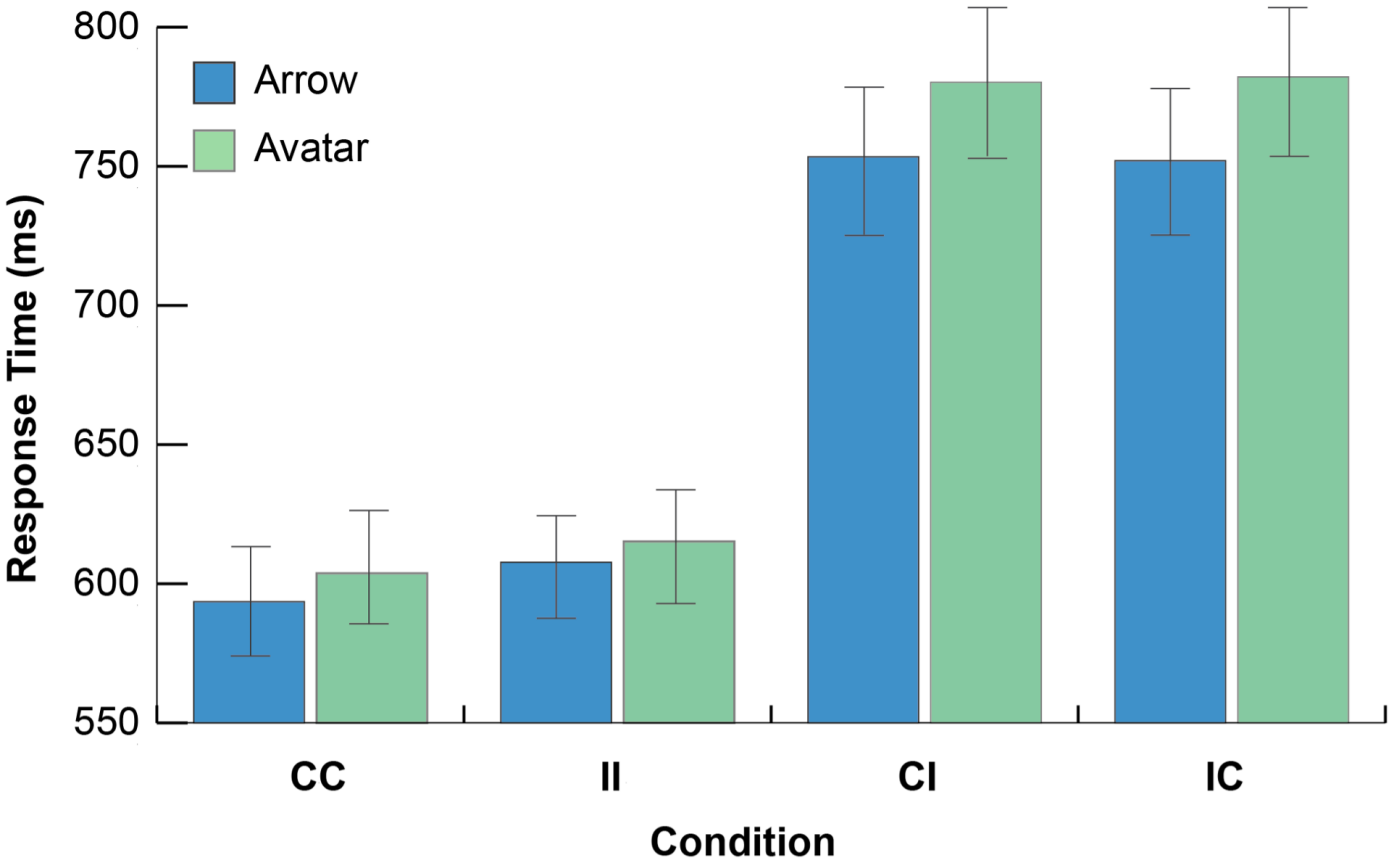
Experiment 1 results. Mean response time as a function of cue type, cue direction consistency, and target content consistency conditions (CC, consistent cue direction/consistent target content; II, inconsistent cue direction/inconsistent target content; CI, consistent cue direction/inconsistent target content; IC, inconsistent cue direction/ consistent target content). Error bars depict the 95% CI.

Mean correct Response time was analyzed using a mixed-effects ANOVA with *Cue Type* (Avatar; Arrow) included as a between-subjects variable, and *Cue Direction Consistency* (Consistent; Inconsistent) and *Target Content Consistency* (Consistent; Inconsistent) included as within-subjects variables. Figure 2 illustrates mean RTs as a function of Cue type, Cue Direction consistency and Target Content consistency.

The analysis indicated that all main effects were significant. A main effect of *Cue Type* (1, 218) =7,789, *p < 0*.0001, MSE = 53,165, *ηp^2^* = 0.973, indicated overall faster responses to targets cued by the arrow than targets cued by gaze. A main effect *Cue Direction Consistency* indicated that overall CC trials were faster than II trials, *F*(1, 218) = 11.87, *p* < 0.001, *MSE* = 788.22, *ηp^2^* = 0.052, which replicates the typical cue directionality gaze following effects [e.g., Friesen and Kingstone (1998); see Capozzi and Ristic (2018) for recent review of this literature]. Finally, a main effect of *Target Content Consistency* indicated that overall consistent mental content between the observer and the cue generated faster response times than inconsistent mental content, *F*(1, 218) = 13.10, *p* < 0.001, *MSE* = 660.19, *ηp^2^* = 0.057.

A two way interaction between *Cue Direction Consistency* x *Target Content Consistency* was significant, *F*(1, 218) = 1214.10, *p* < 0.001, *MSE* = 4748.76, *ηp^2^* = 0.85, and indicated that consistent direction trials were responded to faster when paired with consistent mental content relative to when paired with inconsistent target content [CC vs. CI for arrow: *t*(108) = −24.26, *p* < 0.001; for avatar: *t*(110) = −23.73, *p* < 0.001]. Similarly, inconsistent direction trials were responded to faster when paired with inconsistent mental content relative to when paired with consistent mental content [IC vs. II for arrow: *t*(108) = −21.80, *p* < 0.001; for avatar: *t*(110) = −22.77, *p* < 0.001].

Finally, and supporting our hypothesis, a significant three-way interaction between *Cue Type*, *Cue Direction Consistency*, and *Target Content Consistency*, *F*(1, 218) = 4.48, *p* = 0.035, *MSE* = 4748.76, *ηp^2^* = 0.02, indicated slower performance for the avatar cue relative to the arrow cue for the CI (780 ms avatar vs. 753arrow) and IC (*M* = 782 ms avatar vs. 752 ms arrow) conditions, while there was less of a cue type difference for the CC (604 ms Avatar vs. 594 ms Arrow) and II (615 ms Avatar vs. 608 ms Arrow) conditions. Follow-up independent group t-tests comparing the mean RTs for the Avatar and Arrow cue CI [*t*(218) = 1.48, *p* = 0.14] and IC [*t*(218) = 1.64, *p* = 0.10] conditions did not reach significance. No other effects or interactions were reliable (*Cue Type x Cue Direction Consistency*; *Cue Type x Target Content Consistency*, both *F*s < 1).

### 2.3 Discussion

Experiment 1 data provided proof of concept evidence indicating a performance detriment for targets following dissociated directional and mentalistic aspects of the gaze cue (IC and CI conditions) relative the combined ones (CC and II conditions). Further, and in line with the idea of gaze cues transmitting primarily social information, this behavioral performance detriment was larger when a social avatar served as a cue relative to when a nonsocial arrow served as a cue. Our data also confirmed that participants responded overall faster to targets that are directionally cued (vs. those that are not, demonstrating a classic cueing effect; Friesen and Kingstone, 1998; Frischen et al., 2007) and to those targets with matched mental content. Thus, Experiment 1 results provide one of the first pieces of experimental evidence showing that gaze signals convey information both about *where* the gazer is looking and *what* they are looking at.

However, due to the nature of our design in which they key difference between a social and nonsocial attribution were made based on a between group differences, it remains unknown if those differences reflect differences in social cue processing versus differences between the two groups of participants. To address this issue, and to provide a full replication of our initial finding, in Experiment 2, we ran a preregistered high-powered replication of Experiment 1.

## 3 Experiment 2

Experiment 2 was identical to Experiment 1 except that we recruited new group of participants and manipulated cue type within participants. As before, we hypothesized that if gaze signal includes both directional and mentalistic information, target-related response detriments for dissociated directionality and mental content should be more pronounced when social avatar serves as a cue relative to when a nonsocial arrow serves as a cue.

### 3.1 Methods

The study was pre-registered (osf.io/fwptb). Deidentified data are available at osf.io/3xcqk.

#### 3.1.1 Participants, apparatus, stimuli, design, and procedure

An *a priori* power analysis using an estimated moderate size estimate of *r* = 0.25 for the variability in the magnitude of the gaze cuing with mental state attribution (approximated from Moriguchi et al., 2006; Sulpizio et al., 2015; Tomei et al., 2017) indicated that data from about 95 participants would yield power of 0.8 and data from 130 participants would yield power of 0.9 (Alpha = 0.05).

The data from 136 new participants (114 women, 22 men; mean age = 20.51 years, SD = 1.34) were analyzed^3^. Participants were recruited from the McGill University participant pool and compensated with course credit. All procedures were approved by the University’s Research Ethics Board. Informed consent was obtained from all participants. All participants reported native English fluency, no history of psychiatric or neurological conditions, and normal or corrected-to-normal vision^4^.

Stimuli, apparatus, design, and procedure were identical as in Experiment 1 except that (i) all participants responded to targets cued by the avatar and the arrow cue, (ii) *Cue Type* was blocked, such that half of the blocks presented an avatar cue, and the other half presented the arrow cue. First block assignment was randomized, and subsequent order alternated; (iii), due to an addition of a variable, the total number of trials increased with participants completing eight blocks of 64 trials, for a total of 512 trials. Each block contained 16 trials for each of the four test conditions (CC, II, CI, IC). All conditions were intermixed and presented equally often using a random sequence. Sixteen practice trials with performance feedback were run first. The study was completed in 62.14 min on average (SD = 14.65 min).

### 3.2 Results

Response anticipations (RT < 200 ms) and timeouts (RT > 1800s) accounted for 1.99% of trials and were removed from analyses. When Mauchly’s test was significant, the Greenhouse–Geisser degrees of freedom are reported. All follow-up paired, two-tailed t-tests were Bonferroni corrected. Data were analyzed using SPSS 27.

Overall, the task was well done with 93.03% response accuracy. Mean accuracy was examined for each *Cue Type* (Avatar, Arrow) and *Condition* (CC, II, CI, IC) using a repeated measures ANOVA. The analyses indicated no speed-accuracy trade-off, as the CC (*M* = 0.97) and II (*M* = 0.96) conditions were overall responded to more accurately (and faster, as described in Results) than the CI (*M = 0*.90) and IC (*M* = 0.90) conditions [*Condition* main effect, *F*(2.39, 323.23) = 208.51, *p* < 0.0001, MSE = 0.002, *ηp^2^ = 0.*61] for both cue types. Main effect of *Cue Type* (*F* < 1) and the *Cue Type x Condition* interaction, *F* (2.75,370.99) = 2.63, *p* = 0.055, MSE = 0.001, *ηp^2^ = 0*.019, were not reliable.

To remind, we reasoned that if gaze communicates both directional and mentalistic content, a detriment in target performance should emerge when these two components of a gaze signal are dissociated in the CI and IC conditions. We also predicted that this performance detriment would be more pronounced when the social avatar cue.

**Figure 3:**
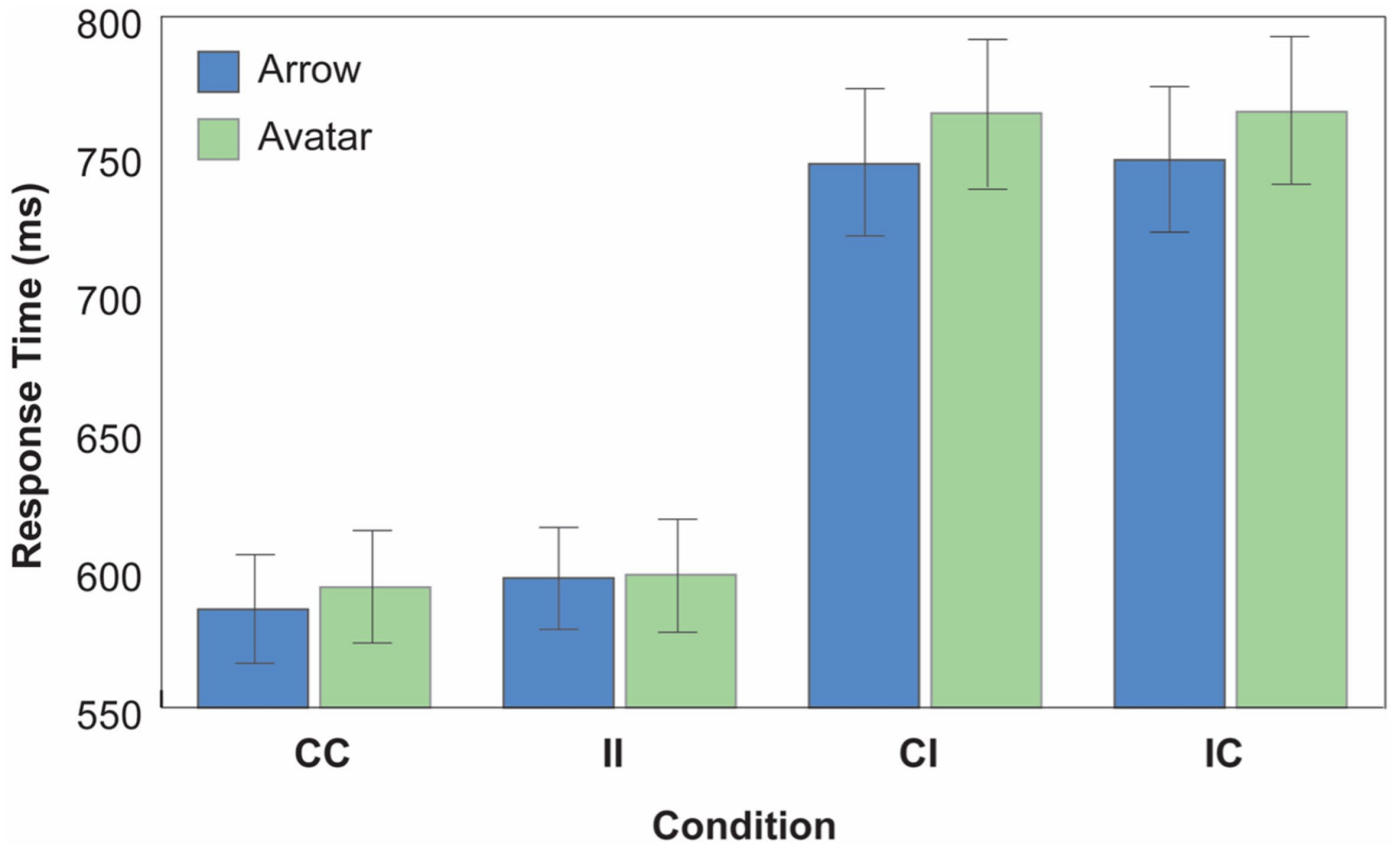
Experiment 2 results. Mean correct response time (RT) as a function of cue type, cue direction consistency and target content consistency conditions (CC, consistent direction/consistent content; II, inconsistent direction/inconsistent content; CI, consistent direction/inconsistent content; IC, inconsistent direction/consistent content). Error bars depict the 95% CI.

A repeated measures ANOVA with *Cue Type* (Avatar; Arrow), *Cue Direction Consistency* (Consistent; Inconsistent), and *Target Content Consistency* (Consistent; Inconsistent) was used to examine mean correct RTs. These means are plotted in Figure 3 as a function of Cue type, Cue Direction consistency, and Target Content consistency conditions.

The results supported our predictions by returning a reliable two-way interaction between *Cue Direction* and *Target Content Consistency* [*F*(1, 135) = 681.68, *p* < 0.001, *MSE* = 10580.12, *ηp2* = 0.835] as well as a reliable three-way interaction between *Cue Type*, *Cue Direction Consistency* and *Target Content Consistency* [*F*(1, 135) = 12.914 *p* < 0.001, MSE = 12111.288, *ηp^2^* = 0.087]. The first interaction indicated that, overall, targets cued by consistent cue direction were responded to faster when that cue direction was paired with consistent target content relative to when it was paired with inconsistent target content [CC vs. CI; Avatar: *t*(135) = −24.92, *p* < 0.001; Arrow: *t*(135) = −22.670, *p* < 0.001]. Likewise, uncued targets (i.e., inconsistent direction trials) were responded to faster when paired with inconsistent target content relative to when paired with consistent target content [II vs. IC; Avatar: *t*(135) = −23.84, *p* < 0.001; Arrow: *t*(135) = −22.25, *p* < 0.001]. This replicates data from Experiment 1.

Further, a significant three-way interaction between *Cue Type*, *Cue Direction Consistency*, and *Target Content Consistency* [*F*(1, 135) = 12.914 *p* < 0.001, MSE = 12111.288, *ηp^2^* = 0.087]. indicated that there was a larger performance difference between the dissociated CI and IC conditions, in which the cue directionality and target mental content are dissociated [CI: *t*(135) = −3.126, *p* = 0.002; IC: *t*(135) = −3.088, *p* = 0.002], when the social avatar served as a cue relative to when a nonsocial arrow served as a cue. In other words, responses to targets following gaze cues were slower than responses to targets following arrow cues in conditions in which directionality and mental content are dissociated. Thus, the dissociation of the two components induced a larger performance determent during avatar trials than during arrow trials. In contrast, there was no difference in response to the targets across the two cue types in the CC and II conditions, where cue directionality and target mental content combined [CC: *t*(135) = −1.756, *p* = 0.081; II: *t*(135) = −0.298, *p* = 0.766; *Cue Type x Cue Direction Consistency*, *F*(1,135) = 1.501, *p* = 0.223]. This replicates our results from Experiment 1, and a well-known finding in the literature showing that social and nonsocial cues typically induce similar directionality effects (Ristic et al., 2002). The interaction between *Cue Type* and *Target Content Consistency* was not reliable (*F* < 1).

As before, the ANOVA returned reliable main effects of *Cue Type* [*F*(1, 135) = 7.59, *p* = 0.007, *MSE* = 4531.75, *ηp^2^* = 0.053] and *Cue Direction Consistency* [*F*(1, 135) = 7.951, *p* = 0.006, *MSE* = 5366.289, *ηp^2^* = 0.056], with overall faster responses to targets cued by the arrow’s direction than targets cued by the avatar’s gaze, and overall faster responses in the CC condition than the II condition across both cue types (i.e., an overall gaze following effect). A main effect of *Target Content Consistency* approached significance, *F*(1, 135) = 3.815, *p* = 0.053, *MSE* = 858.22, *ηp^2^* = 0.027, with trials on which the target content was consistent with the observer’s mental representation generating numerically lower RTs than trials on which the target content representation diverged across the observer and the cue.

Thus, to summarize, in line with our predictions, in Experiment 2, we once again found a reliable detriment in target performance when cue directionality and target mental content (IC and CI conditions) are experimentally dissociated relative to when they are combined (CC and II conditions). Further, and critically, this behavioral performance detriment was larger when a social avatar served as a cue relative to when a nonsocial arrow served as a cue. As such, this result replicated Experiment 1 to once again support the notion that social gaze normally conveys information both about *where* the gazer is looking and *what* they are looking at, i.e., invoking both Level 1 and Level 2 visual perspective processes. When these processes are combined, typical effects of facilitated performance for gaze-directed targets are found. When they are dissociated, a large slowdown in performance is observed.

## 4 General discussion

Grasping the nature of messages conveyed by human gaze is important for understanding the means and the underlying mechanisms of our nonverbal social communication. Using a novel paradigm that measures both combined and dissociated contributions of gazer’s cue directionality and their mental content, in two experiments, here we showed that when these two aspects of the gaze signal are dissociated, significant slowing in performance is found. This is in contrast to the conditions in which cue direction and avatar’s mental content were congruent, where performance was reliably facilitated by such combined representation. Furthermore, the detriment in performance was more pronounced when social avatar delivered the cues. As such, these results are one of the first experimental demonstrations of the influence of both cue directionality *and* mental content in gaze communication and provide a window into the cognitive processes involved in visual perspective taking associated with the Theory of Mind. That is, our findings suggest that the gaze signal typically communicates information about both cue direction and the gazer’s visual perspective, i.e., both *where* agents are looking and *what* they perceive. In other words, understanding of the line of sight depends both on the computation of the direction of the cue and the inferred mental perspective of the gazer. We next bring up and discuss two potential implications of this finding.

First, the typical gaze following response appears to reflect the computation of both the gaze cue’s direction, i.e., the line of sight *and* the representation of the gazer’s mental content. This conclusion follows from our finding showing impaired performance in conditions in which the cue’s directionality and target mental content were dissociated. That is, target performance was slowed in a similar manner by both the absence of the cue’s directional content *and* the absence of the mental representation of the target. Thus, in typical gaze following responses, both cue’s directional information and agents’ mental state appear to be relayed in a joint fashion. When these two processes are experimentally dissociated (i.e., in CI and IC conditions), the gaze following response is slowed. This finding provides an empirical resolution to the longstanding debate in the field of whether gaze conveys directional information or mental content, showing the involvement of both the domain general processes linked to processing of cue directionality as well as domain specific processes linked to computations of social mental content of the gazer (Capozzi and Ristic, 2020).

Dovetailing with this point, our results also showed that dissociating the gaze cue direction information from its mental content signal impacted target performance the most when the social avatar relative to a nonsocial arrow served as a cue. Thus, it seems likely that while the components of cue direction and mental content may be combined in social signals, they appear less intertwined and thus more easily dissociated in nonsocial signals (Marotta et al., 2012). For example, while a simple “line of sight” computation may be easily attributed to a directional cue such as an arrow, this nonsocial stimulus does not possess a mental or visual perspective. That said, while it may be counterintuitive to attribute ‘mental state’ representation to a nonsocial cue, a joint representation of cue directionality and its meaning may still occur in this condition but to a lesser extent given that behaviorally relevant cues like arrows may convey highly meaningful messages in daily life (e.g., Santiesteban et al., 2014). Indeed, recent studies argue that the differences between “social” and “nonsocial” stimuli may reflect both implementation as well as mechanisms differences and could vary with tasks and environmental contextual conditions (Lockwood et al., 2020). The difference between the magnitudes of the dissociated effects between social and nonsocial cues thus may reflect the increased difficulty in disjointing the combined representation of directionality and meaning for social vs. overlearned nonsocial cues (e.g., Ristic and Kingstone, 2012). Future studies are needed to examine how dissociating cue direction from mental content representation may be affected by the differences in the cues’ social and learned values.

More generally, these findings provide significant insights into the cognitive processes associated with Theory of Mind by demonstrating that gaze signals include information on both *where* an agent is looking (directional information) and *what* they are perceiving (mental content). This joint communication maps well onto the two levels of visual perspective-taking. Specifically, the directional component of gaze corresponds to Level 1 perspective-taking, where the observer understands the physical orientation of the gazer’s attention and comprehends that something is within the gazer’s line of sight. In contrast, the mentalistic component aligns with Level 2 perspective-taking, which involves understanding the visual scene from the gazer’s viewpoint, thus requiring a more complex mental state attribution. This implies that Theory of Mind involves not only the ability to infer others’ mental states but also the ability to integrate those inferences with directional information within the environment. Furthermore, our results support the idea that Theory of Mind operates under a hierarchy of cognitive abilities, such that low-level processing of directional cues and higher-level processing of mental states both relate to Theory of Mind processes (Jarrold et al., 2000; Happé and Frith, 2006). Understanding these mechanisms enhances our knowledge of the cognitive processes underlying Theory of Mind, emphasizing a native link between basic directional computation and more involved mental state attribution in social communication (Qureshi and Monk, 2018). Our results suggest that disruptions in these processes may be associated with social cognitive deficits observed in various neurodevelopmental conditions such as autism spectrum disorders, where Theory of Mind tends to be difficult (Rosello et al., 2020; Baron-Cohen et al., 1985; Kimhi, 2014).

Although our work provides valuable new insights about social signals, there are several outstanding points worth considering in future extensions of this work. First, one might wonder if the reported detriment in performance for the dissociated conditions may reflect visual differences in targets, since response targets in the dissociated conditions afford multiple mental representations (i.e., 3 and E) while response targets in the combined condition (i.e., 8) afford a single mental representation. It is important to note that our results showing a larger detriment in performance in dissociated conditions for an avatar relative to an arrow cue occurred across same targets. The same performance detriment also occurred across two experiments and was stable in comparison between and within participants. Thus, while the ease of target representation may overall facilitate performance as indicated in our data, the differences in representation across the two cue types, which are critical for our conclusions, persisted across targets that afford single and multiple mental representations. Nevertheless, it would be important to understand in future work if variability in mental representation of targets influences the contributions of directionality vs. mental content to gaze signals. Second, and dovetailing with this first point, additional future challenge concerns assessments of perspective-taking, given that differentiating between the influences of egocentric and altercentric intrusions, as indices of perspective taking, in tests like these is complex (i.e., Del Sette et al., 2022). Although we aimed to study the impact of cue directionality and mentalistic information on observers’ target judgments, it is possible that egocentric and altercentric intrusions still occurred in our data, where participants made judgments from the avatar’s directional or mental perspective. As a case in point, our data indicated an altercentric intrusion which was evident in the CI condition where observers are spontaneously influenced, i.e., slowed down in responses, by the mismatch between their own and avatar’s visual perspective of target identity. Future studies are needed to understand how assuming self vs. other perspective may influence these attributions and whether the ease of adopting different visual perspectives (e.g., spontaneous vs. explicit) may affect the interpretation of directional and mentalistic aspects of gaze. Finally, it would be important to theoretically link Level-1 and Level − 2 perspective with the respective processing of cue information.

In sum, using a novel behavioral paradigm, across two Experiments, here we show experimentally that that gaze signal conveys both information about *where* an agent is looking and *what* they perceive. This finding provides a new perspective on social signaling by highlighting a complex interplay between the processes involved in contributions of gaze directionality and mental state perception, both of which are highly implicated in the Theory of Mind. As such, this result also opens fruitful new avenues for research on the properties of social gaze communication such as relative contributions of directionality vs. mental content, developmental trajectory and expressions in special groups, variations with individual differences, and/or the underlying neural mechanisms.

## Data Availability

The datasets generated and analyzed for this study can be found in the Open Science Framework directory, osf.io/3xcqk.

## Footnotes

^1^A total of 275 participants completed the study. Participants achieving less than 80% overall accuracy (i.e., over 20% of trials lost due to anticipations, timeouts, or incorrect responses, e.g., McCrackin and Itier, 2019; Van Selst and Jolicoeur, 1994) were excluded from analyses (*n* = 55).

^2^Participants also completed the 28-item Interpersonal Reactivity Index (IRI; Davis, 1983), where, for each question (e.g., “I try to look at everybody’s side of a disagreement before I make a decision”), they responded using a 5-point Likert scale ranging from 1 - “Does not describe me well” to 5 - “Describes me very well.” Exploratory correlation analyses linking individual RT performance in each of the four test conditions (CC, II, CI, IC) suggested that there was a positive relationship between the IRI score and RTs in the II (*r* = 0.189; *p* = 0.047) and CC (*r* = 0.186; *p* = 0.051) conditions in the Avatar group only (Avatar group: 0.111 < *rs* < 0.189, 0.246 < *p* < 0.047; Arrow group: −0.023 < *rs* < −0.059, 0.545 < *p* < 0.808).

^3^A total of 162 undergraduate students completed the study. Data from 26 participants were removed based on the pre-registered exclusion criteria of having less than 80% overall accuracy (i.e., over 20% of trials for each participant lost due to response anticipations, timeouts, or incorrect responses, e.g., McCrackin and Itier, 2019; Van Selst and Jolicoeur, 1994).

^4^As in Experiment 1, participants completed the 28-item Interpersonal Reactivity Index (IRI; Davis, 1983). Exploratory correlation analyses linking individual RT performance in each of the four test conditions (CC, II, CI, IC) with the overall IRI score for yielded no reliable effects (−0.004 < *rs* < −0.088, all *ps* > 0.308).
